# A systematic method for simulating total ionizing dose effects using the finite elements method

**DOI:** 10.1007/s10825-017-1027-2

**Published:** 2017-07-08

**Authors:** Eleni Chatzikyriakou, Kenneth Potter, C. H. de Groot

**Affiliations:** Department of Electronics and Computer Science, University of Southampton, University Road, Southampton, SO17 1BJ UK

**Keywords:** Total ionizing dose, Carrier transport, Finite elements method, Synopsys, MOS

## Abstract

Simulation of total ionizing dose effects in field isolation of FET technologies requires transport mechanisms in the oxide to be considered. In this work, carrier transport and trapping in thick oxides using the finite elements method in the Synopsys Sentaurus platform are systematically simulated. Carriers are generated in the oxide and are transported out through a direct contact with the gate and thermionic emission to the silicon. The method is applied to calibrate experimental results of 400 nm $\hbox {SiO}_{2}$ capacitors irradiated at total doses of 11.6 kRad ($\hbox {SiO}_{2}$) and 58 kRad ($\hbox {SiO}_{2}$). Drift–diffusion-enabled trapping as well as other issues that arise from the involved physics are discussed. Effective bulk trap densities and activation energies of the traps are extracted.

## Introduction

Thick field isolation is widely used today as a Buried OXide (BOX) in silicon-on-insulator technologies [1], as shallow trench isolation separating devices in single and multi-gate technologies [2] and it is also commonly found in 2D semiconductor transistors such as graphene and $\hbox {MoS}_{2}$ [3, 4]. These oxides are the main contributing factor to Total Ionizing Dose (TID) effects of deep submicron technologies, contrary to the thin gate oxides, where trapped charges are able to tunnel out under the influence of the electric field [5].

Three-dimensional finite elements method (FEM) simulation of TID in transistor models is generally avoided due to the complexity introduced in its use. Experimental work is often complemented by the ‘fixed oxide charge’ method. Using this method, unrealistic results have been reported such as in cases where the electric field produces non-uniform distribution of charge [6]. Much work on 2D analytical modeling of TID in shallow trench isolation regions of MOSFETs has been performed previously [7, 8]. However, only a 3D model could accurately describe the effects of the charges gathering in the various locations in the field oxides, inducing leakage paths in the device. In [9], TID simulations using the FEM simulator Florida Object-Oriented Reliability Simulator (FLOORS) are presented. In this work, we present the methodology to simulate TID effects using the commercial simulation software Synopsys Sentaurus. Sentaurus operation is analogous to that of FLOORS; namely, it solves partial differential equations for the operation of devices built using process simulation. However, it is more powerful, as various models can be combined, and the system can be examined in a technology-to-circuit manner.

We have shown three-dimensional FEM simulations of TID in 22-nm bulk FinFET devices in [10]. We present here the methodology used to this end. System calibration is performed on experimental results of 400 nm $\hbox {SiO}_{2}$ buried oxide capacitors. The method is based on taking into account the drift motion of the carriers for the trapping process, as was described in detail by Leray et al. [11–13]. The method can be extended to include hydrogen transport and trap formation at the interface of the oxide with the silicon [14, 15] using state transitions that are implemented in the software through a physical model interface. This allows for an elaborate and comprehensive treatment of TID effects in state-of-the-art FET technologies for use in radiation harsh environments that can also be combined with other reliability effects [16].

## Simulation models

The physics models used in the simulations are:

- Gamma radiation, by which electron–hole pairs are generated,
- Basic trapping equations with SRH recombination,
- Drift–diffusion carrier transport in the oxide,
- Thermionic emission at the Si/oxide interface

These are described in detail in this section, and the procedure used to derive their parameters is explained.

### Carrier transport

Carriers are generated in the oxide using the simple equation,

1 $$\begin{aligned} G_{\mathrm {ehp}} = \dot{D} \cdot g_{\mathrm {SiO_2}} \cdot f_{\mathrm {y}}\left( E\right) \end{aligned}$$

with $\dot{D}$, the dose rate (rad($\text {SiO}_{\text {2}})/\text {s}$), $f_{\mathrm {y}}$ the carrier yield and $\hbox {g}_{\mathrm{SiO_2}}$ is a material-dependent irradiation constant set to $7.6 \cdot 10^{12}\ \hbox {ehp}\times \hbox {cm}^{-3} \times \hbox { Rad}(\hbox {SiO}_{2})^{-1}$ [10].

**Fig. 1:**
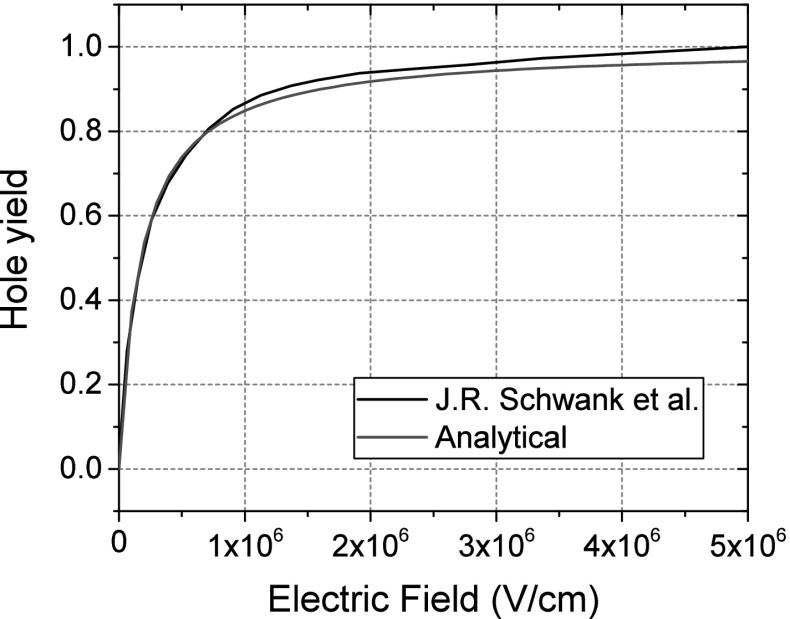
Hole yield as a function of electric field. The simulation parameters were fitted to $\hbox {Co}^{60}$ from [17]

The electron–hole pair yield, ${f}_{\mathrm{y}}$, is given as

2 $$\begin{aligned} f_{\mathrm {y}} \left( E\right) =\left( \frac{| E | +E_{{1}}}{| E | +E_{{2}}} \right) ^m \end{aligned}$$

where *E* is the electric field (V/cm). The irradiation experiments in this work were performed using a $\hbox {Co}^{60}$ source; therefore, the fitting parameters are ${E}_{1} = 0.1\hbox { V/cm}, E_{2} = 2\times 10^{5}\hbox { V/cm}$ and m = 0.9, following the work by [17] where the carrier yield as a function of electric field was recorded (Fig. 1).

Carriers escape the oxide through either the gate electrode or thermionic emission at the Si/$\hbox {SiO}_{2}$ heterointerface. The Sentaurus device implementation of the thermionic emission model assumes that electron energy and transverse momentum are conserved during the transition through the interface and takes into account transmission and reflection [18, 19]. Equilibrium conditions are preserved on both sides of the interface.

Only donor traps are included in the trapping simulations. Therefore, Poisson’s equation in the oxide takes the form,

3 $$\begin{aligned} \nabla \cdot \left( \epsilon \nabla \phi \right) = - q \left( p - n + N_{\mathrm {D}}^{{+}} - N_{\mathrm {A}}^{\mathrm {-}} + p_{\mathrm {t}}^{{+}} \right) \end{aligned}$$

where *q* is the electron charge, $N_{\text {D}}^{\text {+}}$ and $N_{\text {A}}^{{-}}$ are ionized donor and acceptor impurities respectively, and $p_{\text {t}}^{{+}}$ is the density of trapped holes ($\text {cm}^{-3}$).

The electron and hole continuity equations that include carrier generation and trap-assisted recombination take the following form in transient simulations [20, 21]:

4 $$\begin{aligned} \frac{\partial n}{\partial t}= & {} \frac{1}{q}\nabla \cdot \mathrm {J}_n + G_{\mathrm {ehp}}-R_{\mathrm {nt}}^n \end{aligned}$$

5 $$\begin{aligned} \frac{\partial p}{\partial t}= & {} -\frac{1}{q}\nabla \cdot \mathrm {J}_p + G_{\mathrm {ehp}}-R_{\mathrm {pt}}^p \end{aligned}$$

where $J_{n,p}$ are the electron and hole current densities (A/$\hbox {cm}^{2}$) which are calculated in this work using the classical drift–diffusion model. Mobility values take the previously established values of 20 $\hbox {cm}^{2}$/Vs for electrons and $10^{-5} \hbox { cm}^{2}$/Vs for holes [21].

### Carrier trapping

Studies using electron paramagnetic resonance and electron spin resonance techniques have pinpointed primary defects in quartz and $\alpha$-$\hbox {SiO}_{2}$ that trap positive carriers. They are denoted as $E'$-centers: $E{1}'$ in quartz and $E_{\gamma }'$ and $E_{\delta }'$ in $\alpha$-$\hbox {SiO}_{2}$ [22–24].

Oxygen vacancies are at the heart of these defects. The $E_{\gamma }'$ center has been found to be a deep hole trap, having an anisotropic spin distribution. It is thought to be an oxygen vacancy that undergoes asymmetric relaxation. In the neutral state, Si atoms nearby oxygen vacancies bond to form a dimer. In the positively charged state, one of these Si atoms relaxes and bonds with a fourth oxygen atom [24–26]. There is no consensus over the nature of the $E_{\delta }'$ center. It is a shallow hole trap. Prevailing theories are that it is either single oxygen vacancy that remains in the dimer state in both the charged and neutral states [27] or a cluster of four vacancies of $E_{\gamma }'$-like dangling bonds [28–33].

The occupational probability, $f_p$, of a trap is used here to describe the state of the trap. This is a number between 0 and 1 which, when multiplied by the number of trapping sites, gives the number density of the occupied states and therefore properly accounts for the charged state Coulomb potential. It is given by,

6 $$\begin{aligned} p_{\mathrm {t}}^{{+}} = p_{\mathrm {t}} \times f_p \end{aligned}$$

The occupational probability depends on capture and emission processes for the traps as follows,

7 $$\begin{aligned}&p+p_{\mathrm {t}} \Leftrightarrow p_{\mathrm {t}}^{{+}} \end{aligned}$$

8 $$\begin{aligned}&n+p_{\mathrm {t}}^{{+}} \Leftrightarrow p_{\mathrm {t}} \end{aligned}$$

The rate of change of the hole occupational probability is described using the capture and emission rates of the trap,

9 $$\begin{aligned} \frac{\partial f^p}{\partial t} = \left( 1-f^p\right) \left( e_{\mathrm {CB}}^n+c_{\mathrm {VB}}^p\right) - f^p \left( c_{\mathrm {CB}}^n+e_{\mathrm {VB}}^p\right) \end{aligned}$$

There are four capture and emission rates in the hole occupational probability equation. These are emission of an electron to the conduction band, $e_{\mathrm {CB}}^{n}$ (trapping), emission of a hole to the valence band, $e_{\mathrm {VB}}^{p}$ (de-trapping), capture of an electron from the conduction band, $c_{\mathrm {CB}}^{n}$ (de-trapping) and capture of a hole from the valence band, $c_{\mathrm {VB}}^{p}$ (trapping).

From collision theory, capture events are defined similarly to the inverse of the Shockley–Read–Hall carrier lifetimes, namely capture rate = carrier density $\times$ capture cross section $\times$ carrier velocity ($\hbox {s}^{-1}$). For the emission rates, carrier density is substituted by the effective carrier densities for SRH recombination, $n_{{1}}$ for electrons and $p_{{1}}$ for holes [11, 20],

10 $$\begin{aligned} e_{\mathrm {CB}}^{n}= & {} v_{\mathrm {th}}^n\sigma _n n_1 \end{aligned}$$

11 $$\begin{aligned} e_{\mathrm {VB}}^{p}= & {} v_{\mathrm {th}}^p\sigma _p p_1 \end{aligned}$$

where $v_{\text {th}}^n$ and $v_{\text {th}}^p$ are the thermal velocities of electrons and holes (cm/s), $\sigma _n$ and $\sigma _p$ the capture cross sections of electrons and holes ($\text {cm}^{\text {2}}$).

For the capture events, the simple capture rate equation suffices in the case where trapping is dominated by diffusion of the carriers in the oxide. However, to properly represent the capture events, the drift motion of the carriers is also included in the equations by introducing the probability factor $j_{\mathrm{coef}}$ [34].

12 $$\begin{aligned} c_{\mathrm {CB}}^{n}= & {} \sigma _n\left[ \left( 1-j_{\mathrm {coef}}\right) v_{\mathrm {th}}^nn+j_{\mathrm {coef}}\frac{J_n}{q}\right] \end{aligned}$$

13 $$\begin{aligned} c_{\mathrm {VB}}^{p}= & {} \sigma _p\left[ \left( 1-j_{\mathrm {coef}}\right) v_{\mathrm {th}}^pp+j_{\mathrm {coef}}\frac{J_p}{q}\right] \end{aligned}$$

For example, with $j_{\text {coef}}=1$, trapping is dependent on the current density, which is described in our calculations using the drift–diffusion Sentaurus device model, but with $j_{\text {coef}}=0$, it is only dependent on the carrier densities and their thermal velocities.

The effective carrier densities are given by,

14 $$\begin{aligned} n_1= & {} N_{\mathrm {C}}\mathrm {exp}\left\{ \frac{q\times \left( E_{\mathrm {trap}}-E_{\mathrm {C}} \right) }{kT}\right\} \end{aligned}$$

15 $$\begin{aligned} p_1= & {} N_{\mathrm {V}}\mathrm {exp}\left\{ \frac{q\times \left( E_{\mathrm {V}}-E_{\mathrm {trap}}\right) }{kT}\right\} \end{aligned}$$

with $E_{\text {trap}}, E_{\text {C}}, E_{\text {V}}$ the activation energy of the trap, the conduction and the valence band, respectively (eV), $N_{\text {C}}$ and $N_{\text {V}}$ ($\text {cm}^{{-3}}$) are the effective densities of states of the conduction and valence bands.

The recombination processes for one trapping level are,

16 $$\begin{aligned} R_{\mathrm {pt}}^n= & {} p_{\mathrm {t}}\left[ \left( 1-f_p\right) c_{\mathrm {CB}}^n-f_pe_{\mathrm {CB}}^n\right] \end{aligned}$$

17 $$\begin{aligned} R_{\mathrm {pt}}^p= & {} p_t\left[ f_pc_{\mathrm {VB}}^p-\left( 1-f_p\right) e_{\mathrm {VB}}^p\right] \end{aligned}$$

In quasi-stationary, the electron and hole recombination rates become equal and for SRH recombination the equation becomes,

18 $$\begin{aligned} R_{\mathrm {SRH}} = \frac{p_t\left[ c_{\mathrm {CB}}^n c_{\mathrm {VB}}^p - \left( c_{\mathrm {CB}}^nc_{\mathrm {VB}}^pn_{\mathrm {i,eff}}^2\right) /np\right] }{e_{\mathrm {CB}}^n+c_{\mathrm {CB}}^n+e_{\mathrm {VB}}^p+c_{\mathrm {VB}}^p} \end{aligned}$$

where $n_{\text {i,eff}}$ is the intrinsic carrier density in the oxide ($\text {cm}^{{-3}}$).

## Pre-irradiation calibration

**Fig. 2:**
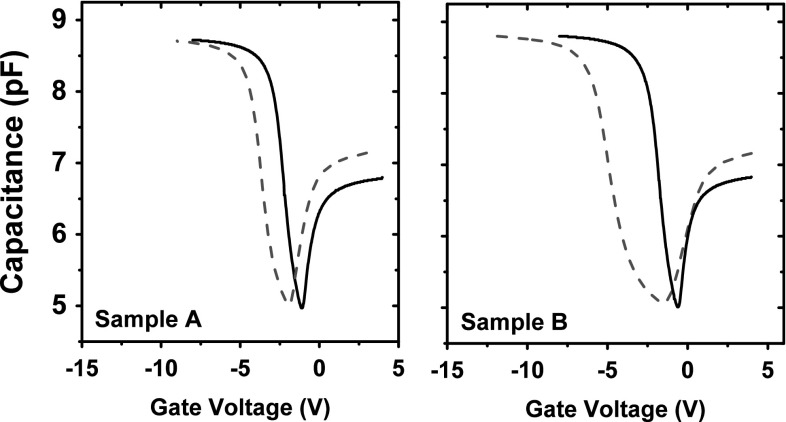
C–V results of 400 nm $\text {SiO}_{\text {2}}$ capacitor samples: Sample A irradiated at 11.6 kRad ($\text {SiO}_{\text {2}}$) and Sample B irradiated at 58 kRad ($\text {SiO}_{\text {2}}$) [35]. *Black lines* showing pre-rad and *dashed red line* showing post-rad results (Color figure online)

$\text {SiO}_{\text {2}}$ BOX capacitors with 400 nm oxide thickness were fabricated using commercial Smart-Cut^®^ wafers at the University of Southampton. They were subsequently irradiated using $\text {Co}^{\text {60}}$ source at a dose rate of 38.6 Rad($\text {SiO}_{\text {2}}$)/s. Two total doses were used: Sample A with 11.6 kRad ($\text {SiO}_{\text {2}}$) and Sample B with 58 kRad($\text {SiO}_{\text {2}}$). The fabrication and characterization process is explained in detail in [35].

Experimental pre- and post-irradiation characteristics of the capacitor are shown in Fig. 2. The C–V measurements were taken using the Agilent 4155C parameter analyzer at 1 MHz AC frequency. Depletion/inversion region capacitance increase occurs when the AC signal is too low compared to the equilibration rate of the mobile carriers in the depletion region. In this case, however, the AC signal is adequately high to prevent inversion charges to gather at the Si/$\hbox {SiO}_{2}$ interface. This rare occasion of inversion characteristics is due to a lateral AC current that extends beyond the metallic plate of the gate and increases the measured capacitance [35, 36]. This effect has not been examined further in this work as it is not affected by irradiation.

**Fig. 3:**
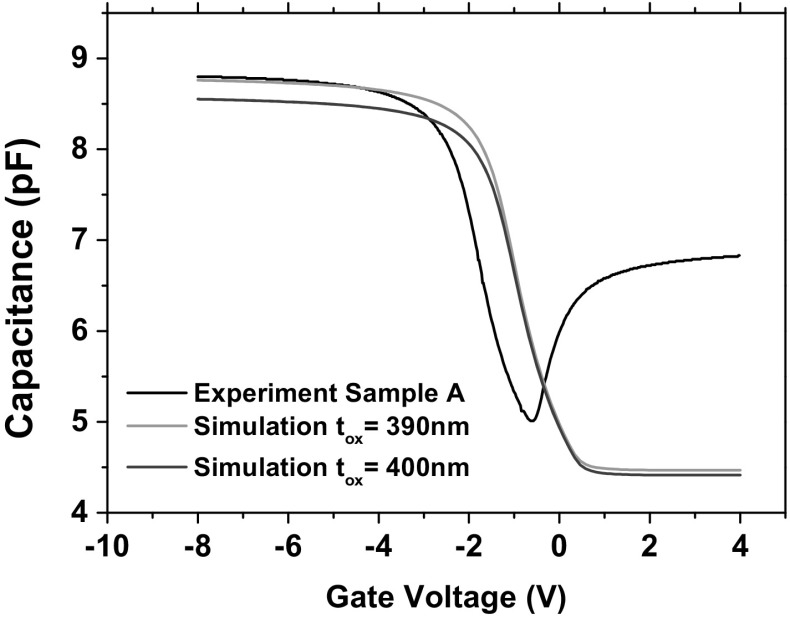
Calibration of simulated results. Experimental results for Sample A are compared to simulation results with different oxide thickness. The simulations did not include fixed charges in the capacitor oxides

**Fig. 4:**
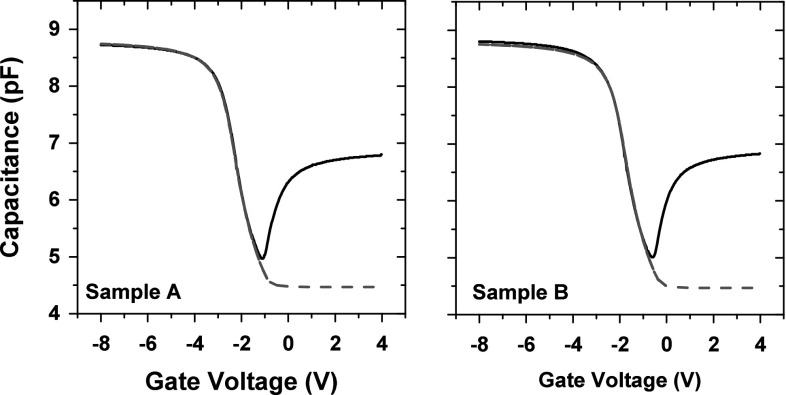
Pre-rad C–V results of Samples A and B. *Black line* indicating experimental and *red dashed line* simulation results (Color figure online)

A 2D simulation model was constructed in Sentaurus TCAD. The capacitor model was calibrated to the experimental pre-rad results by using a doping concentration $N_{\text {a}} = 6.4\times \text {10}^{\text {14}}\ \text {cm}^{{-3}}$, oxide thickness $\text {t}_{\text {ox}} = 390\,\hbox {nm}$ and metal work function of 4.28  eV. Simulated C–V results for different capacitor oxide thickness are compared to the experimental results in Fig. 3. It is observed that the fabrication process has already introduced positive fixed oxide charges that have shifted the C–V curve to negative voltages. Therefore, fixed oxide charge ($N_{\text {ot}}$) was also introduced in the simulation model to fit the midgap voltage to the experiment. For Sample A $N_{\text {ot}} = \text {6}\times \text {10}^{\text {15}}$ $\text {cm}^{{-3}}$ and for Sample B $N_{\text {ot}} = \text {4.5}\times \text {10}^{\text {15}}\ \text {cm}^{{-3}}$. Pre-rad simulation and experimental results are shown in Fig. 4.

**Fig. 5:**
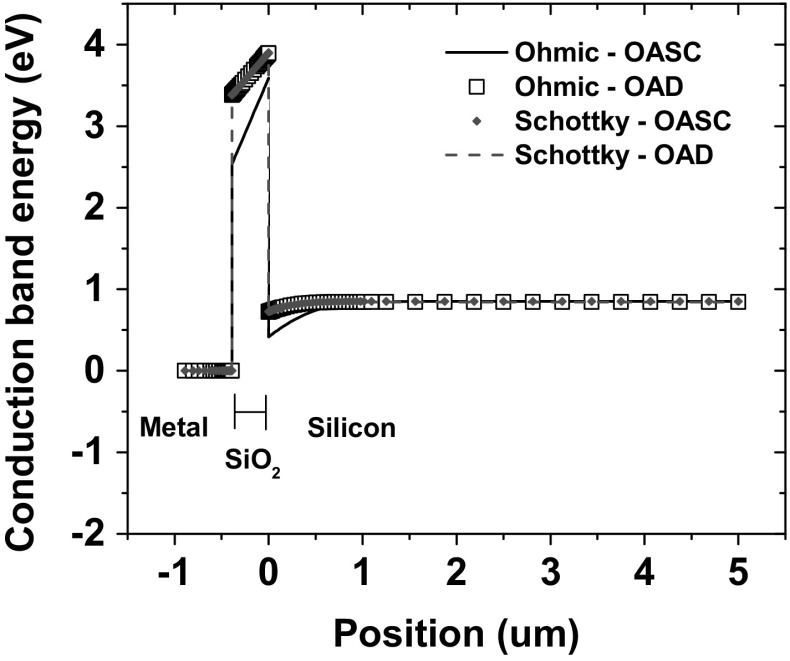
Band diagram of the capacitor showing the conduction band edge and electron Fermi level with the oxide as semiconductor (OASC) and as dielectric (OAD) with Schottky and ohmic metal contact

Metal–insulator transitions are a subject of intense interest in the fields of solid-state physics and materials science [37, 38]. In this work, we have followed a simple approach to simulate carrier transport mechanisms and metal/insulator interface physics in the context of solution of the differential equations in the FEM simulator. The $\hbox {SiO}_{2}$ region was defined to be a semiconductor. The contact of the oxide with the metal was defined as Schottky in order to properly account for the metal/semiconductor interface physics [39]. Figure 5 shows the conduction band energy for the cases of the oxide defined to be a dielectric (OAD) and a semiconductor (OASC). The band bending in the case of an ohmic contact with the OASC material produces unwanted shifts in the device characteristics.

## Trapping model parametrization

Parameters used for the trapping model are shown in Table 1. Cross-section values as derived in [12] are used. Simulations showed no change in the final results for values up to two orders of magnitude higher.

Carrier transport parameters in $\hbox {SiO}_{2}$, such as effective masses and Density of States (DOS), have been thoroughly examined in [40, 41]. In our configuration, the DOS for electrons and holes becomes important only for very shallow traps and does not affect simulation results otherwise. This stems from the use of the effective DOS in the emission terms (10) and (11). Particularly, the hole emission term, which reflects trapped hole annealing, causes reduced trapping rates at trap energies $\approx$1 eV above the valence band and shallower.

**Table 1:** Simulation parameters used for hole trapping in the oxide

Description	Symbol	Value	Unit
Electron capture cross section	$\sigma _{{n}}$	$\text {6.8}\times \text {10}^{{-14}}$	$\text {cm}^{\text {2}}$
Hole capture cross section	$\sigma _{{p}}$	$\text {10}^{{-12}}$	$\text {cm}^{\text {2}}$
Electron thermal velocity	$\text {v}_{\text {th}}^{\text {n}}$	$\text {2.042}\times \text {10}^{\text {7}}$	cm/s
Hole thermal velocity	$\text {v}_{\text {th}}^{\text {p}}$	$\text {1.562}\times \text {10}^{\text {7}}$	cm/s
Conduction band DOS	${N}_{\mathrm{C}}$	$10^{18}$–$10^{20}$	$\hbox {cm}^{-3}$
Valence band DOS	${N}_{\mathrm{V}}$	$10^{18}$–$10^{20}$	$\hbox {cm}^{-3}$
Effective hole trap energy	${E}_{\mathrm{trap}}$	${E}_{\mathrm{V}}+4$	eV

**Fig. 6:**
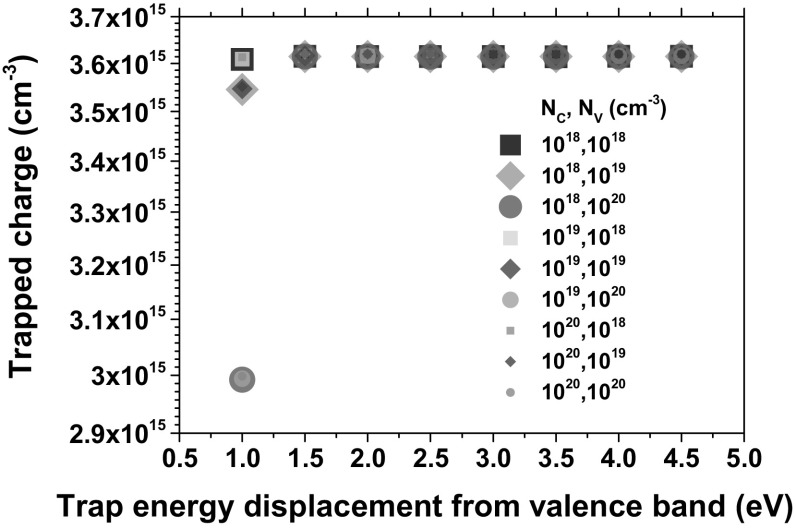
Average trapped charge in the oxide after $D=11.6$ kRad ($\hbox {SiO}_{2}$) as a function of ${E}_{\mathrm{trap}}$ for different DOS values

To visualize this, simulations with various DOS values were performed for increasing trap energy, as shown in Fig. 6. The average trapped charge was extracted from the device simulation results using Sentaurus Visual. The total dose used was 11.6 kRad ($\hbox {SiO}_{2}$). At ${E}_{\mathrm{trap}}= {E}_{\mathrm{V}} + 1\hbox { eV}$, the trapped charge decreases as the DOS value increases. This is more pronounced for the valence DOS. The final trapped charge remains constant in all other cases.

A number of studies have been made on $\hbox {SiO}_{2}$ defects using density functional theory calculations. For the dimer configuration (E$_\delta '$ center), the shallow activation energy of 1 eV from the valence band edge is found. This configuration is found to exist in densities of $\approx$80% while the bistable defect (E$_\gamma '$ center) with activation energy of 4.5 eV at a concentration of $\approx$20%. Further studies have shown that immediately after irradiation, the predominant trapping site is the shallow E$_\delta '$, and gradually, as the shallow traps get annealed with time, charges get trapped in deep hole trapping sites [32, 42].

**Fig. 7:**
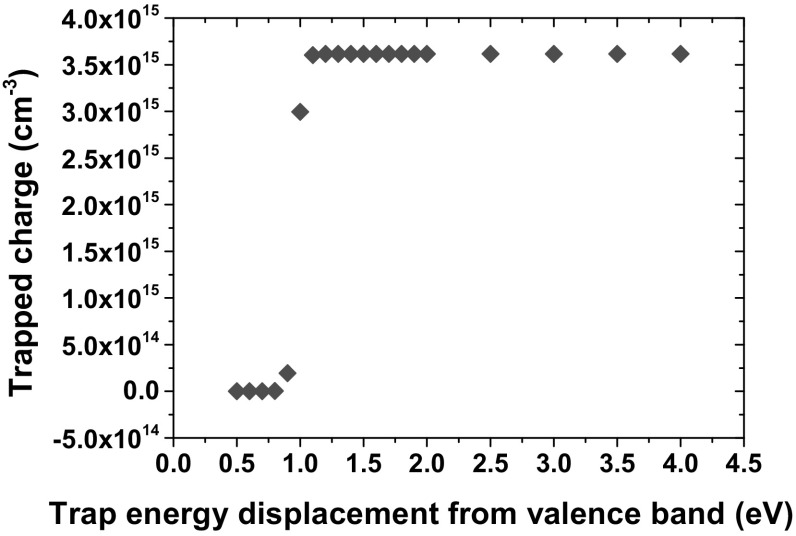
Average trapped charge in the oxide for increasing ${E}_{\mathrm{trap}}$ after $D=11.6$ kRad ($\hbox {SiO}_{2}$) with ${N}_{\mathrm{C}} = 10^{19}\hbox { cm}^{-3}$ and ${N}_{\mathrm{V}}=10^{20}\hbox { cm}^{-3}$

The change in trapped charge for a wider range of ${E}_{\mathrm{trap}}$ values is also shown in Fig. 7. For very shallow traps, hole trap annealing dominates the trapping mechanism. Only deep traps of energy ${E}_{\mathrm{V}}+4\mathrm{eV}$ were used in further simulations, in order to account for long-term trapping effects.

**Fig. 8:**
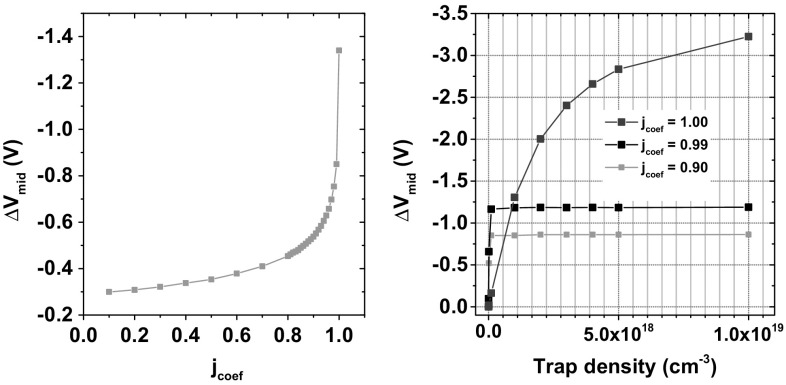
Midgap threshold voltage shift with bulk trap density $10^{18} \hbox { cm}^{-3}$ as a function of $j_{\mathrm{coef}}$ (*left*). Midgap voltage shift with increasing trap density for three different configurations of $j_{\mathrm{coef}}$ (*right*)

Charge trapping under low electric field is dominated by carrier diffusion and by drift under higher electric field [7]. Low electric field is encountered when the device is unbiased, or under situations of electric field collapse due to space charge effects [11]. The contributions of such processes are controlled using the $j_{\mathrm{coef}}$ as described in (12) and (13). Despite the fact that our device was unbiased during irradiation, it was observed that trapping was best described using the drift–diffusion carrier transport model included in Sentaurus device and was unrealistic when a contribution of carrier concentration of even 1% was introduced.

This is shown in Fig. 8. The midgap voltage shift increases exponentially with $j_{\mathrm{coef}}$. When seen as a function of trapped charge, $\Delta {V}_{\mathrm{mid}}$ is cutoff abruptly at $j_{\mathrm{coef}}=0.99$. Furthermore, the highest value of $\Delta {V}_{\mathrm{mid}}$ in this case is lower than the post-irradiation voltage shift observed experimentally for this total dose, up to a trap density of $10^{19}\hbox { cm}^{-3}$. Therefore, it is observed that, only the equations that take into account the drift–diffusion current transport model at a percentage of 100% can adequately represent the experimental data.

Both the Schottky contact and the thermionic emission model assured a correct description of the trapping effects at the boundaries of the oxide. Band bending at the ohmic metal/semiconductor contact created unrealistic pre-irradiation voltage shifts, while thermionic emission eliminated the high density of trapped charge at the Si/$\hbox {SiO}_{2}$ interface due to electric field discontinuities resulting from the conduction band difference between the two regions.

## Post-irradiation calibration

**Fig. 9:**
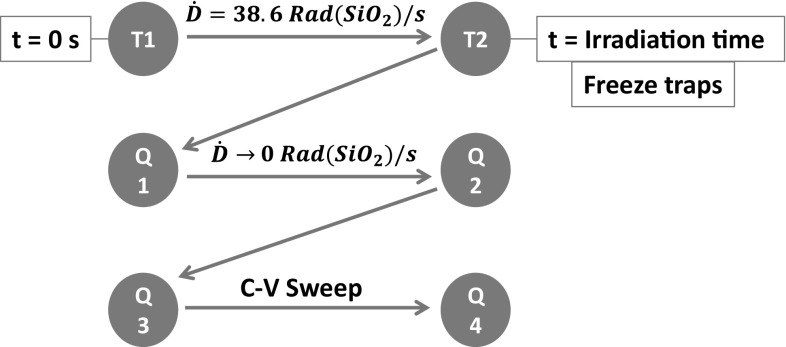
Simulations flow for post-rad results. ‘T’ indicates beginning and ending of transient run. ‘Q’ indicates beginning and ending of quasi-stationary run

The simulation flow for the device irradiation is shown in Fig. 9. Post-rad simulations were performed in transient (indicated with ‘T’ ) first and subsequently ‘freezing’ the state of the traps, to account for un-annealed charge in quasi-stationary C–V (indicated with ‘Q’ ).

**Fig. 10:**
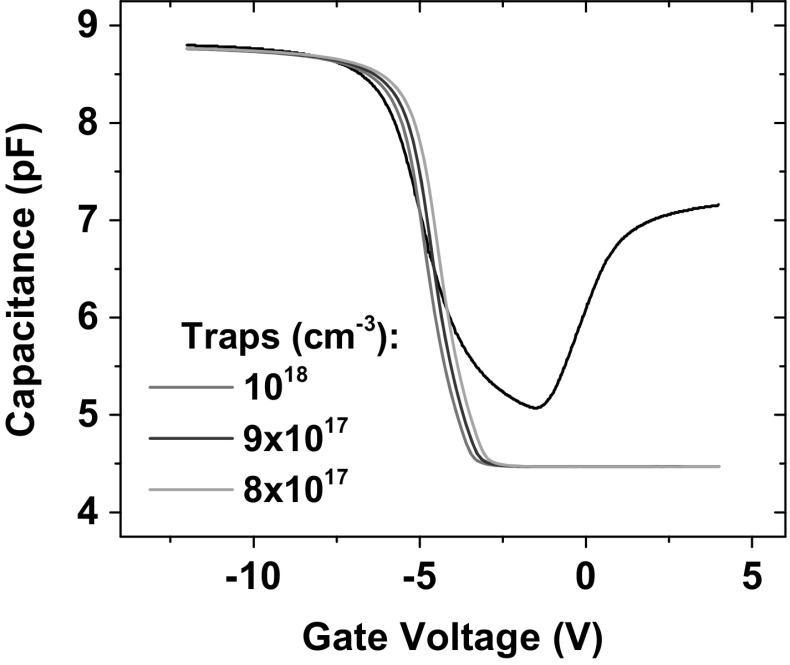
Calibration of post-rad simulation results to experimental results (Sample B). The trap density is chosen by keeping the ionizing dose constant

The ionizing dose was kept constant to the value used during the experiments, a uniform hole trap density was introduced in the oxide and varied as shown in Fig. 10.

**Fig. 11:**
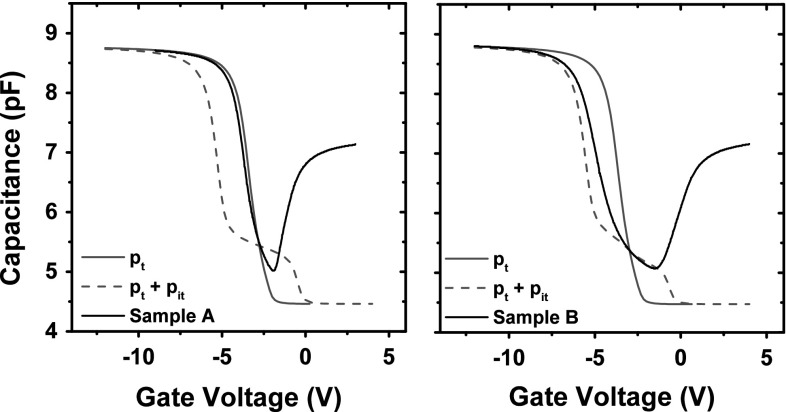
Calibration to midgap voltage shift. ${p}_{\mathrm{t}}$ denotes the C–V results with bulk oxide traps. ${p}_{\mathrm{t}}+{p}_{\mathrm{it}}$ denotes bulk oxide traps and interface traps. *Black solid line* denotes experimental results

The following method was used to calibrate the simulation post-rad results to the midgap voltage of the experimental capacitors: Donor and acceptor interface traps with equal densities and activation energies of 0.56 eV above and below the conduction band, respectively, were defined. The midgap voltage was then found from the intersection between the experimental C–V curve and the point where the simulation C–V curve crosses as shown in Fig. 11. This point represents the ‘intrinsic energy’ (${E}_{\mathrm{i}}$) in the middle between ${E}_{\mathrm{C}}$ and ${E}_{\mathrm{V}}$. With this computational method for determining the midgap voltage, errors resulting from analytical extraction methods were avoided.

In Fig. 11, flatband and threshold voltage shifts are also observed on top of $\Delta {V}_{\mathrm{mid}}$. This indicates the creation of interface traps which results from transport of hydrogen species originating from the oxide and other parts of the device and de-passivating interface dangling bonds [14, 43, 44].

**Fig. 12:**
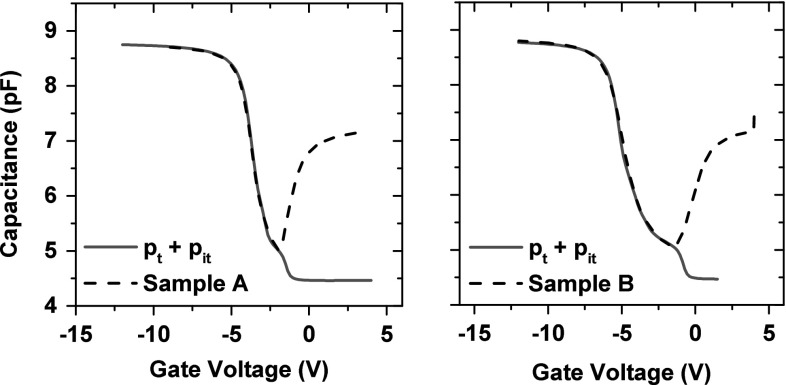
Calibration of post-irradiation results with bulk oxide traps and interface traps

The final device post-rad characteristics are shown in Fig. 12. Densities of the bulk and interface traps are shown in Table 2. Average interface trap densities are derived for interface states with different activation energies. The samples in this study were measured one day after irradiation, and therefore, bulk trap densities are lower than found previously in commercial thick oxide capacitors [12].

**Table 2:** Bulk and interface trap densities

Types of traps	Sample A	Sample B	Unit
Bulk (donors)	$8.2\times 10^{17}$	$5\times 10^{17}$	$\hbox {cm}^{-3}$
Interface donors	$5.2\times 10^{9}$	$2.0 \times 10^{10}$	${\mathrm{eV}}^{-1}\hbox {cm}^{-2}$
Interface acceptors	$2.0\times 10^{10}$	$5.0 \times 10^{10}$	$\mathrm{eV}^{-1}\hbox {cm}^{-2}$

**Fig. 13:**
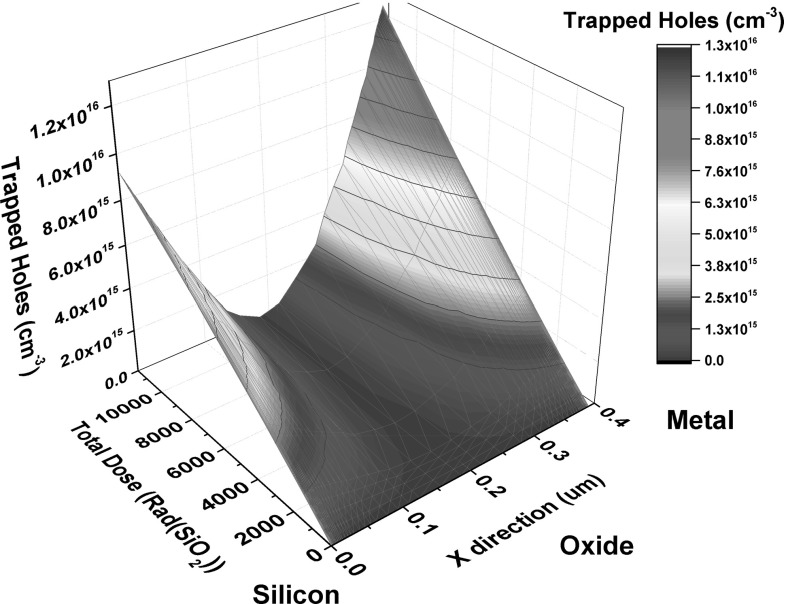
Trapped charge density in a horizontal 1D cut in the middle of the oxide region as a function of total dose

The distribution of trapped charge in the oxide for a 1D cut at the middle of the capacitor is shown in Fig. 13. With increasing radiation, the holes are trapped in greater numbers toward the interfaces of the oxide with the silicon and the metal gate. This process is repeated until the system reaches equilibrium. Steady-state conditions are difficult to be achieved as the holes generated in the oxide change direction based on the trapping and annealing mechanisms taking place. The electric field in the middle of the oxide is also reduced dramatically as a result of the hole trapping at the interfaces. This also reduces generation of carrier pairs in that region [11].

The distribution of the trapped holes becomes important when enabling ionic hydrogen transport ($\hbox {H}^{+}$) in the oxide, as the trapped holes can potentially create an electrostatic fence for the $\hbox {H}^{+}$ to move toward the interface and thus reducing trap formation density. This has been postulated to occur in high dose rate regimes in [45].

Overall, the subject of interface trap formation has many microscopic properties that are still under investigation. In Sentaurus device, hydrogen species transport as well as state transitions can be used to investigate their dynamics and expand the present model.

## Conclusions

A FEM simulation methodology was presented for the examination of TID effects in MOS structures by solving charge transport and trapping equations in the oxides. The oxides are treated as semiconducting structures from which carriers are able to escape through either a Schottky contact with the metal or thermionic emission with the silicon. This assured unrealistic trapping of carriers at the oxide boundaries was avoided. Calibration of the system to experimental results in $\hbox {SiO}_{2}$ capacitors revealed effective trap densities of $5 \times 10^{17}\hbox { cm}^{-3} - 8.2 \times 10^{17}\hbox { cm}^{-3}$.

This method is applicable for field isolation in FETs where the majority of the effects result from holes trapped in the bulk of the oxide. Use of process simulation can be made for examination of even more complex devices allowing for a full treatment of TID effects in state-of-the-art transistor technologies and memory cells. The method can be extended to include interface trap formation from reactions of mobile ionic hydrogen with interface dangling bonds. The TID mechanism in Sentaurus device can also be combined with other reliability models, such as single event upsets and variations in device characteristics from different sources to examine their combined contribution in the technologies of interest.
